# Twice the Joy, but Added Risk: Exploring Twin Birth Outcomes at the East Suffolk and North Essex NHS Foundation Trust Maternity Service, England

**DOI:** 10.7759/cureus.119171

**Published:** 2026-11-05

**Authors:** Aakanksha, Phyo Ei Mon, Arushi Sharma, FNU Sunita, Johnbosco E Mamah

**Affiliations:** 1 Obstetrics and Gynaecology, East Suffolk and North Essex NHS Foundation Trust, Colchester, GBR

**Keywords:** birthweight discordance, cesarean section, chorionicity, nhs, postpartum hemorrhage, preterm birth, twin pregnancy

## Abstract

Background and aim

Twin pregnancy carries higher maternal and neonatal risk than singleton pregnancy, and local data on chorionicity and national benchmarking remain limited. This study primarily aims to describe maternal characteristics, timing and mode of birth, and maternal and neonatal outcomes of twin deliveries in a two-site NHS maternity service. Secondary, hypothesis-generating aims were to explore factors associated with cesarean birth and early delivery and to make an indicative comparison with UK national audit data.

Materials and methods

We conducted a retrospective, descriptive, and analytical cross-sectional study of 55 women who delivered twins (110 fetuses) between October 2, 2025, and June 18, 2026, following STROBE and RECORD guidance. Key proportions are presented with 95% CIs. Timing of birth was assessed against chorionicity-specific National Institute for Health and Care Excellence (NICE) windows. Analyses were exploratory and unadjusted, and no formal comparison with national data was made.

Results

Mean maternal age was 31.8 ± 5.7 years. Of the 55 pregnancies, 46 (83.6%) were dichorionic diamniotic (DCDA), nine (16.4%) were monochorionic diamniotic (MCDA), and 10 (18.2%) followed assisted conception. Mean gestation at delivery was 36.1 ± 1.6 weeks; 33 women (60.0%) delivered before 37 weeks. Compared with chorionicity-specific NICE windows, 24 of 46 DCDA pregnancies (52.2%) delivered before 37+0 weeks, and seven of nine MCDA pregnancies (77.8%) before 36+0 weeks. Cesarean birth of both twins occurred in 42 women (76.4%; 95% CI 63.7-85.6): 24 (43.6%) elective and 18 (32.7%) unscheduled. Postpartum hemorrhage (≥500 mL after vaginal birth or ≥1000 mL after cesarean) occurred in 19 women (34.5%), and composite maternal morbidity in 18 (32.7%). Mean birthweight was 2417 ± 457 g; nine of 53 pairs (17.0%) had ≥20% birthweight discordance. At least one twin was admitted to the neonatal unit (any level of care) in 20 of 54 pregnancies (37.0%), including four of four (100%) born before 34 weeks. One fetus (one of 110, 0.9%) died in utero at 23+3 weeks; there were no neonatal or maternal deaths.

Conclusions

Preterm birth, cesarean birth, postpartum hemorrhage, and neonatal unit admission were common in this twin population. With 55 women, CIs were wide, so the study cannot show whether local outcomes differ from national data. Early delivery of MCDA twins relative to the NICE window and the reasons recorded for cesarean birth warrant local review. Structured documentation of the planned mode of birth, the reasons for the decision, cesarean urgency, neonatal level of care, and indications for admission would support more reliable audits.

## Introduction

Multiple pregnancy accounts for about one in 65 pregnancies in Great Britain and has remained near 15 per 1000 for two decades [1]. In 2023, the National Maternity and Perinatal Audit (NMPA) identified 8301 women who gave birth to twins, triplets, or quadruplets, confirming that twin care remains routine in NHS maternity services [1].

Twin pregnancy is associated with increased risks for both women and their babies [2,3]. Compared with singleton pregnancy, it is associated with substantially higher maternal morbidity, including potentially life-threatening conditions and near-miss events [2], higher rates of pre-eclampsia [3], and increased risk of stillbirth and neonatal death [4].

Clinicians must decide when and how twins should be born [5-8]. A systematic review reported that the prospective risk of stillbirth rises after 37+0 weeks in dichorionic twins and after 36+0 weeks in monochorionic diamniotic (MCDA) twins [5], and NICE recommends planned birth from these gestations in uncomplicated pregnancies [6]. The Twin Birth Study randomized 2804 women with a cephalic first twin at 32+0 to 38+6 weeks; death or serious neonatal morbidity was similar with planned cesarean and planned vaginal birth (2.2% vs 1.9%), although 43.8% of the planned vaginal group had a cesarean [7]. A Cochrane review found no evidence to support routine planned cesarean for term twins with a cephalic first twin [8]. Nationally, about 18% of women with twins had a vaginal birth (13.3% unassisted and 4.55% instrumental), whereas planned and unplanned cesarean accounted for 42.9% and 36.5% of births, respectively [1].

Local review is constrained by data quality. Chorionicity, which guides surveillance and timing, was coded for fewer than 7% of multiple-birth records in the English maternity dataset in 2023, and neither planned mode of birth nor method of conception is collected nationally [1]. After the trust implemented its electronic patient record in October 2025, we reviewed all twin deliveries recorded during its first nine months. The primary objective was descriptive: to report maternal characteristics, timing and mode of delivery, and maternal and neonatal outcomes. Secondary objectives were exploratory and hypothesis-generating: to examine factors associated with cesarean birth and with delivery before the chorionicity-specific recommended window, to describe neonatal unit admission by gestational age at delivery, and to make an indicative comparison with UK national audit data and published evidence. These analyses identify associations only and do not establish causation.

## Materials and methods

Design and setting

This retrospective, descriptive, and analytical cross-sectional study adheres to the STROBE statement for cross-sectional studies [9] and its extension for routinely collected health data (RECORD) [10]. The setting was the two-site maternity service (Colchester and Ipswich hospitals) of East Suffolk and North Essex NHS Foundation Trust (ESNEFT). The trust information team identified cases through an ad hoc report from the electronic patient record (EPIC) listing every woman with a twin delivery (two births recorded in the delivery episode) at either site between October 2, 2025, and June 18, 2026.

The study population therefore comprised 55 women with twin pregnancies, representing 110 fetuses. Because cases were identified from deliveries at the trust, pregnancies that ended before 24 weeks (miscarriage or termination), higher-order multiple pregnancies, and women transferred antenatally to give birth at another unit were not captured. Therefore, the study team had no denominator of twin pregnancies at risk, and the total number of births in the period was unavailable. Because the hospital site was not identified, data from both sites were analyzed together. In this article, “women” includes everyone who gives birth. The project was registered with the trust as a service evaluation, and the Health Research Authority decision tool indicated that research ethics committee review was not required [11]. Only members of the clinical team accessed the data, which were held on trust systems in accordance with the UK General Data Protection Regulation, the Data Protection Act 2018, and the trust information governance policy, and were reported only in aggregate. As a service evaluation of routinely collected data, the project did not require individual consent, and no identifiable information is reported.

Participants and data

The authors abstracted clinical data from the full electronic record of all 55 women using a standardized 33-field pro forma (Appendix 1) covering maternal characteristics, chorionicity, pregnancy complications, delivery details, blood loss, and neonatal outcomes and complications. Each record was abstracted once, without duplicate extraction or formal inter-rater assessment. Internal checks involved recalculating birthweight discordance from recorded birthweights, comparing the calculated and recorded values, and assessing sex concordance in monochorionic pairs; discrepancies were corrected after reviewing the record. Gestational age was taken as recorded in the electronic record. National practice dates pregnancies by first-trimester crown-rump length, or by embryo transfer date after in vitro fertilization, but the dating method was not recorded in the dataset. Estimated blood loss (EBL) was taken as recorded; whether it was measured or visually estimated was not recorded. Missing data were handled by complete-case analysis for each variable, without imputation, and the denominator is shown wherever data were incomplete. Follow-up was limited to the birth admission and any readmission documented in the record. Because total births were unavailable, the twinning rate was not calculated. Umbilical cord pH was recorded as a single value per infant, and the source records did not state whether samples were arterial or venous. Neonatal unit admission was recorded without the level of care (intensive, high-dependency, or special care) or the indication for admission. Only the first-listed indication for cesarean birth was available, and the planned mode of birth was not recorded.

Definitions

Preterm birth was defined as delivery before 37+0 weeks. Because NICE recommends planned birth from 37+0 weeks for uncomplicated dichorionic diamniotic (DCDA) twins and from 36+0 weeks for uncomplicated MCDA twins [6], timing was also assessed against these chorionicity-specific windows: delivery before 37+0 weeks (DCDA) or before 36+0 weeks (MCDA) was classified as before the recommended window, and delivery at 37+0 to 37+6 weeks (DCDA) or 36+0 to 36+6 weeks (MCDA) as within it. A pregnancy was classified as uncomplicated if none of the following was recorded: growth restriction or abnormal Doppler (as defined below), hypertensive disorder, gestational diabetes, rupture of membranes before 37 weeks, placental abruption, obstetric cholestasis, structural fetal anomaly, polyhydramnios, twin-to-twin transfusion syndrome, or fetal death. All other pregnancies were classified as complicated. Previous cesarean, malpresentation, and maternal conditions such as treated hypothyroidism or asthma did not, by themselves, make a pregnancy complicated.

Cesarean urgency followed the four-category classification [12], with elective cesarean section recorded as ELCS (category 4) and unscheduled cesarean section as EMCS (categories 1 to 3). The main comparison was the cesarean birth of both twins versus the vaginal birth of the first twin, including forceps and sequential births in the vaginal delivery group. Postpartum hemorrhage was defined using conventional mode-specific thresholds of EBL: ≥500 mL after vaginal birth and ≥1000 mL after cesarean birth. EBL was also categorized as ≥1000 mL (major hemorrhage) [13] and ≥1500 mL, the threshold reported by the National Maternity and Perinatal Audit [1]. Composite maternal morbidity comprised EBL ≥1000 mL, blood transfusion, or intensive or high-dependency care; a composite was used because each component was too infrequent to analyze separately. Growth restriction was defined using a composite measure of antenatally documented growth concerns. It was assigned retrospectively, during data abstraction after delivery, from the antenatal record or the documented indication for delivery, and was not derived from birthweight. It comprised explicitly documented fetal or selective growth restriction (n = 7), abnormal umbilical artery Doppler without a documented diagnosis of growth restriction (n = 3), placental dysfunction (n = 1), and small for gestational age recorded as the indication for delivery (n = 1). Birthweight discordance was calculated as $\frac{\mathrm{heavier} - \mathrm{lighter}}{\mathrm{heavier}} \times 100$, using 20% and 25% thresholds [1]. Neonatal unit admission required documented neonatal unit or neonatal intensive care unit admission at any level of care, and a low Apgar score was <7 at five minutes. Preterm births were classified from the documented indication as spontaneous (spontaneous preterm labor or prelabor rupture of membranes) or provider-initiated (all others). Fetal deaths were classified using UK definitions: a baby born at or after 24+0 weeks showing no signs of life is a stillbirth, but a baby confirmed to have died before 24 weeks is not registered as a stillbirth, even if delivered later [14].

Statistical analysis

Categorical data are reported as numbers and percentages (n (%)); continuous data are reported as mean ± SD or median (IQR). 95% CIs for key proportions were calculated using the Wilson method. Proportions were compared using Fisher’s exact test. Odds ratios (OR) are conditional maximum likelihood estimates with exact 95% CIs and require no correction for zero cells; when a cell is zero, the OR cannot be estimated, and only the exact CI is shown. Because the two-sided Fisher p-value and the exact CI are calculated differently, they can disagree when p is close to 0.05, and such results are regarded as inconclusive. The 95% CI for the paired birthweight difference was based on the t distribution. The pregnancy was the unit of analysis for all comparisons; infant-level outcomes are reported descriptively only, and birthweights within pairs were compared with a paired test, so the non-independence of twins did not affect the statistical tests. Continuous variables were compared between groups using the Mann-Whitney U test, correlations were assessed using Spearman's rank correlation coefficient, and the birthweights of the first and second twins were compared using a paired t-test. All tests were two-sided (p < 0.05). Given this sample size and number of comparisons, all inferential results are exploratory, hypothesis-generating, and unadjusted for multiple testing. No formal statistical comparison with national data was made. Analyses were conducted in Python 3 using SciPy (Python Software Foundation, Wilmington, DE, USA).

Comparison with published evidence

Comparators were restricted to the UK and other high-income settings: the latest UK national audit of multiple births [1], a Norwegian national registry study of hypertensive disorders in twin pregnancy [3], and the largest randomized trial of mode of twin birth with its Cochrane synthesis [7,8]. We identified sources through targeted searches of online bibliographic, guideline, and audit resources in September 2026. This is an indicative, structured narrative comparison, not a PRISMA-compliant systematic review; no meta-analysis or formal statistical comparison was conducted.

## Results

Study population

The study population comprised 55 women with twin pregnancies, representing 110 fetuses: 109 live births (99.1%) and one intrauterine fetal death (0.9%), diagnosed at 23+3 weeks and delivered with its liveborn co-twin at 37+5 weeks (Figure 1). Because death was confirmed before 24 weeks, this loss is classified as a fetal loss before 24 weeks (late miscarriage) and not as a stillbirth, although MBRRACE-UK asks for such deaths to be reported for surveillance [14]. Because neonatal fields were blank for one delivery, neonatal analyses included 54 of 55 pregnancies (98.2%) and 107 liveborn infants.

**Figure 1:**
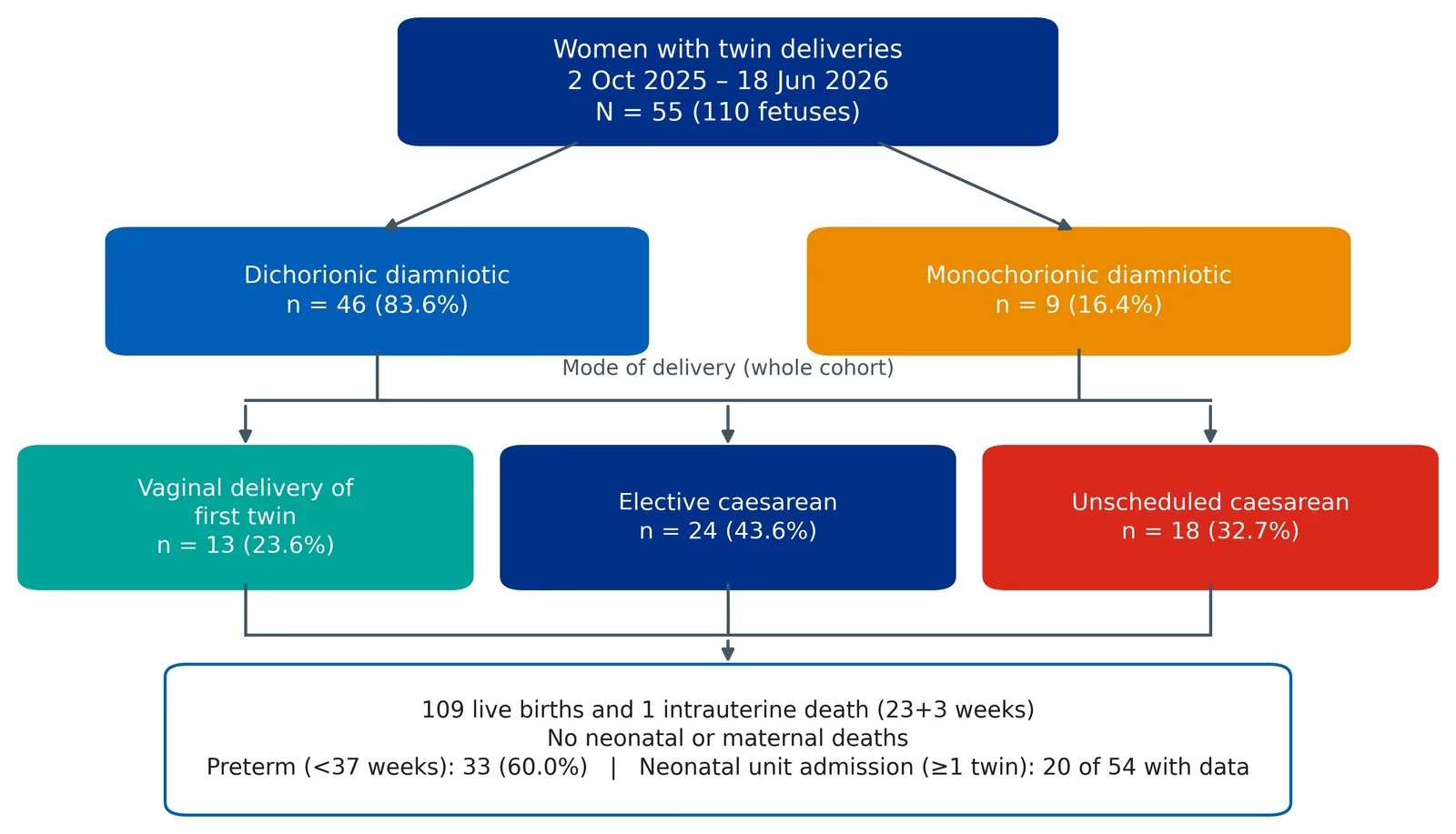
Study cohort (N = 55): chorionicity, mode of delivery, and summary outcomes

Maternal characteristics

The 55 women had a mean age of 31.8 ± 5.7 years; 12 (21.8%) were 35 or older, and five (9.1%) were 40 or older. Twenty-three women (41.8%) were nulliparous, nine (16.4%) had a prior cesarean, and 10 (18.2%) conceived with assistance. Booking body mass index (BMI) was recorded for 37 women (67.3%), of whom 14 (37.8%) had a BMI of at least 30 kg/m². Chorionicity was documented for all pregnancies: 46 were DCDA (83.6%) and nine (16.4%) were MCDA (Table 1).

**Table 1:** Maternal demographic and obstetric profile (N = 55) No woman had a BMI below 18.5 kg/m². BMI: body mass index, IVF: in vitro fertilization, IUI: intrauterine insemination, SD: standard deviation, DCDA: dichorionic diamniotic, MCDA: monochorionic diamniotic

Characteristic	Value
Age (years)	
Mean ± SD	31.8 ± 5.7
Median (range)	31 (21-50)
<25, n (%)	3 (5.5)
25-29, n (%)	17 (30.9)
30-34, n (%)	23 (41.8)
35-39, n (%)	7 (12.7)
≥40, n (%)	5 (9.1)
Parity and history, n (%)	
Nulliparous	23 (41.8)
Para 1	20 (36.4)
Para ≥2	12 (21.8)
Previous cesarean	9 (16.4)
Booking BMI, kg/m², n (%) (n = 37 recorded)	
18.5-24.9	9 (24.3)
25.0-29.9	14 (37.8)
≥30	14 (37.8)
≥35 (subset of ≥30)	5 (13.5)
Ethnic group, n (%)	
White (including three recorded as Romanian)	41 (74.5)
Black	8 (14.5)
Asian	5 (9.1)
Mixed	1 (1.8)
Conception and smoking, n (%)	
Assisted conception (total)	10 (18.2)
IVF	9 (16.4)
IUI followed by IVF	1 (1.8)
Current smoker (n = 53 recorded)	5 (9.4)
Chorionicity, n (%)	
DCDA	46 (83.6)
MCDA	9 (16.4)

Antenatal complications

Antenatal complications were common: hypertensive disorders and gestational diabetes each affected nine women (16.4%), and five women (9.1%) had pre-eclampsia; rupture of membranes before 37 weeks affected six (10.9%); and growth restriction, placental dysfunction, or abnormal Doppler affected 12 pregnancies (21.8%). One MCDA pregnancy (1.8%) had twin-to-twin transfusion syndrome with type 2 selective growth restriction, and one DCDA pregnancy (1.8%) had intrauterine death of one twin at 23+3 weeks. Nineteen pregnancies (34.5%) met the definition of uncomplicated and 36 (65.5%) were complicated (Table 2).

**Table 2:** Antenatal maternal and twin-specific complications (N = 55; more than one per pregnancy possible) Maternal anemia and placenta praevia were not recorded in the final dataset.

Complication	n (%)
Maternal	
Hypertensive disorder	9 (16.4)
Of which pre-eclampsia	5 (9.1)
Of which severe pre-eclampsia	2 (3.6)
Gestational diabetes	9 (16.4)
Rupture of membranes before 37 weeks	6 (10.9)
Hypothyroidism	4 (7.3)
Placental abruption (confirmed or suspected)	2 (3.6)
Asthma	2 (3.6)
Obstetric cholestasis	1 (1.8)
Cervical cerclage and/or vaginal progesterone	8 (14.5)
Twin-specific	
Growth restriction / placental dysfunction / abnormal Doppler	12 (21.8)
of which explicit fetal or selective growth restriction	7 (12.7)
Twin-to-twin transfusion syndrome	1 (1.8)
Structural anomaly or variant in ≥1 twin	3 (5.5)
Polyhydramnios	3 (5.5)
Intrauterine death of one twin	1 (1.8)
Summary	
Uncomplicated pregnancy (see Definitions)	19 (34.5)
Complicated pregnancy	36 (65.5)

Gestation at delivery

Gestation at delivery ranged from 29+5 to 38+2 weeks (mean 36.1 ± 1.6; median 36+2). Thirty-three women (60.0%; 95% CI 46.8-71.9) delivered before 37 weeks, and four (7.3%) before 34 weeks (Table 3). Because planned birth is recommended from 36+0 weeks for MCDA twins, delivery before 37 weeks is expected under the recommended timing of birth, so timing was also assessed against chorionicity-specific windows. Overall, 31 women (56.4%) delivered before their recommended window, 23 (41.8%) within it, and one (1.8%) after it. Seven of nine MCDA pregnancies (77.8%) delivered before 36+0 weeks and 24 of 46 DCDA pregnancies (52.2%) before 37+0 weeks. The median gestational age at delivery was 35+0 weeks for MCDA pregnancies versus 36+5 weeks for DCDA pregnancies (Figure 2). Based on the documented indication, eight of the 33 preterm births (24.2%) followed spontaneous preterm labor or prelabor rupture of membranes, and 25 (75.8%) were provider-initiated or had no documented spontaneous onset; because onset of labor was not a structured field, some spontaneous births may have been misclassified (Table 3). Delivery before the recommended window occurred in 26 of 36 complicated pregnancies (72.2%) and five of 19 uncomplicated pregnancies (26.3%).

**Table 3:** Gestation, presentation, and mode of delivery (N = 55) SD: standard deviation, IQR: interquartile range, ELCS: elective lower-segment cesarean section, EMCS: emergency cesarean section, DCDA: dichorionic diamniotic, MCDA: monochorionic diamniotic, NICE: National Institute for Health and Care Excellence, PPROM: preterm prelabor rupture of membranes

Variable	n	%
Gestation at delivery		
Mean ± SD, weeks	36.1 ± 1.6	
Median (IQR), weeks+days	36+2 (35+1 to 37+2)	
<32+0 weeks	1	1.8
32+0 to 33+6 weeks	3	5.5
34+0 to 36+6 weeks	29	52.7
≥37+0 weeks	22	40.0
Preterm (<37+0 weeks)	33	60.0
Preterm after documented spontaneous labor or PPROM	8	14.5
Preterm, provider-initiated or onset not documented	25	45.5
Timing against chorionicity-specific NICE window		
Before window (DCDA <37+0; MCDA <36+0 weeks)	31	56.4
Within window (DCDA 37+0 to 37+6; MCDA 36+0 to 36+6 weeks)	23	41.8
After window	1	1.8
Presentation		
First twin cephalic	38	69.1
First twin breech	16	29.1
First twin transverse	1	1.8
Second twin non-cephalic	27	49.1
Both twins cephalic	20	36.4
Mode of delivery		
Vaginal, both twins, no instruments	11	20.0
Forceps delivery	1	1.8
Vaginal first twin, cesarean second twin	1	1.8
Elective cesarean (ELCS or category 4)	24	43.6
Unscheduled cesarean (EMCS or category 1–3)	18	32.7
Cesarean for both twins (total)	42	76.4

**Figure 2:**
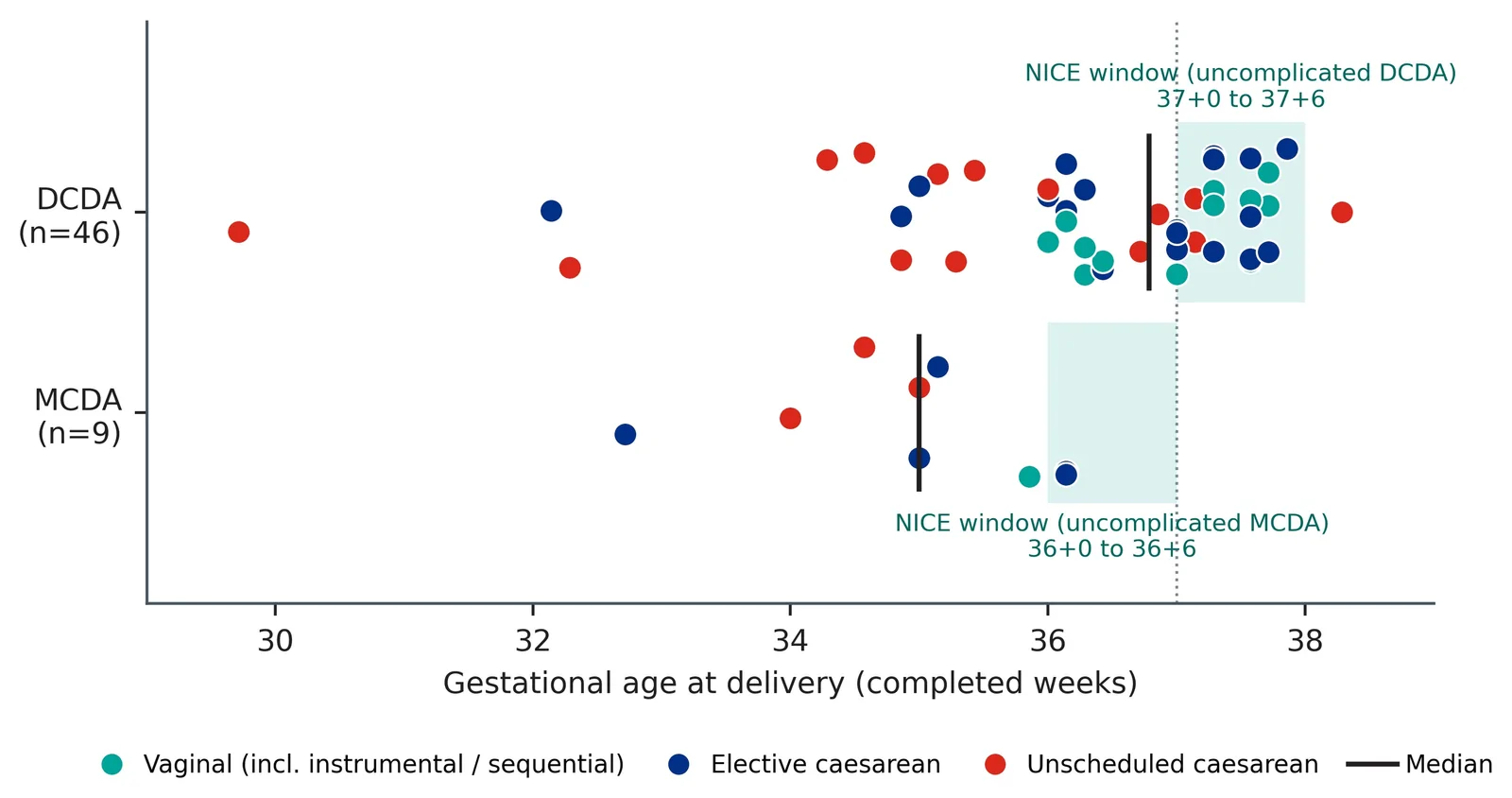
Gestational age at delivery by chorionicity and mode of delivery. Shaded bands show NICE-recommended windows for uncomplicated pregnancies; most pregnancies here had at least one recorded complication Each symbol represents one pregnancy (N = 55). Solid vertical lines show the median gestational age in each group, and the dashed line marks 37 weeks. NICE: National Institute for Health and Care Excellence, DCDA: dichorionic diamniotic, MCDA: monochorionic diamniotic

Of the seven MCDA pregnancies delivered before 36+0 weeks, two followed spontaneous preterm labor or prelabor rupture of membranes (one with twin-to-twin transfusion syndrome and type 2 selective growth restriction at 32+5 weeks and one at 34+4 weeks), two followed acute maternal or placental complications (suspected placental abruption at 34+0 weeks and severe pre-eclampsia at 35+0 weeks), and two were for abnormal fetal Doppler findings (at 35+0 and 35+6 weeks). One was a planned cesarean at 35+1 weeks for a breech first twin in an otherwise uncomplicated pregnancy. The two MCDA pregnancies delivered within the window were both delivered at 36+1 weeks.

Among the 19 uncomplicated pregnancies, 13 (68.4%) delivered within the NICE-recommended window, five (26.3%) before it, and one (5.3%) after it. Of the five delivered before the window, three had unscheduled cesareans (breech first twin; labor after a previous cesarean with a breech first twin; abnormal cardiotocography), and two had planned cesareans (maternal request at 36+3 weeks; breech first twin in an MCDA pregnancy at 35+1 weeks). One DCDA pregnancy was delivered at 38+2 weeks by unscheduled cesarean after failed induction of labor.

Presentation and mode of delivery

In 38 pregnancies (69.1%), the first twin was cephalic; in 16 (29.1%), breech; and in one (1.8%), transverse. Cesarean birth of both twins occurred in 42 women (76.4%; 95% CI 63.7-85.6): 24 elective (43.6%) and 18 unscheduled (32.7%). Thirteen women (23.6%) delivered the first twin vaginally, including one forceps delivery (1.8%) and one sequential cesarean for the second twin (1.8%) (Table 3, Figure 3). Median gestation was lower for cesarean than for vaginal delivery (36+1 versus 37+0; p = 0.042).

**Figure 3:**
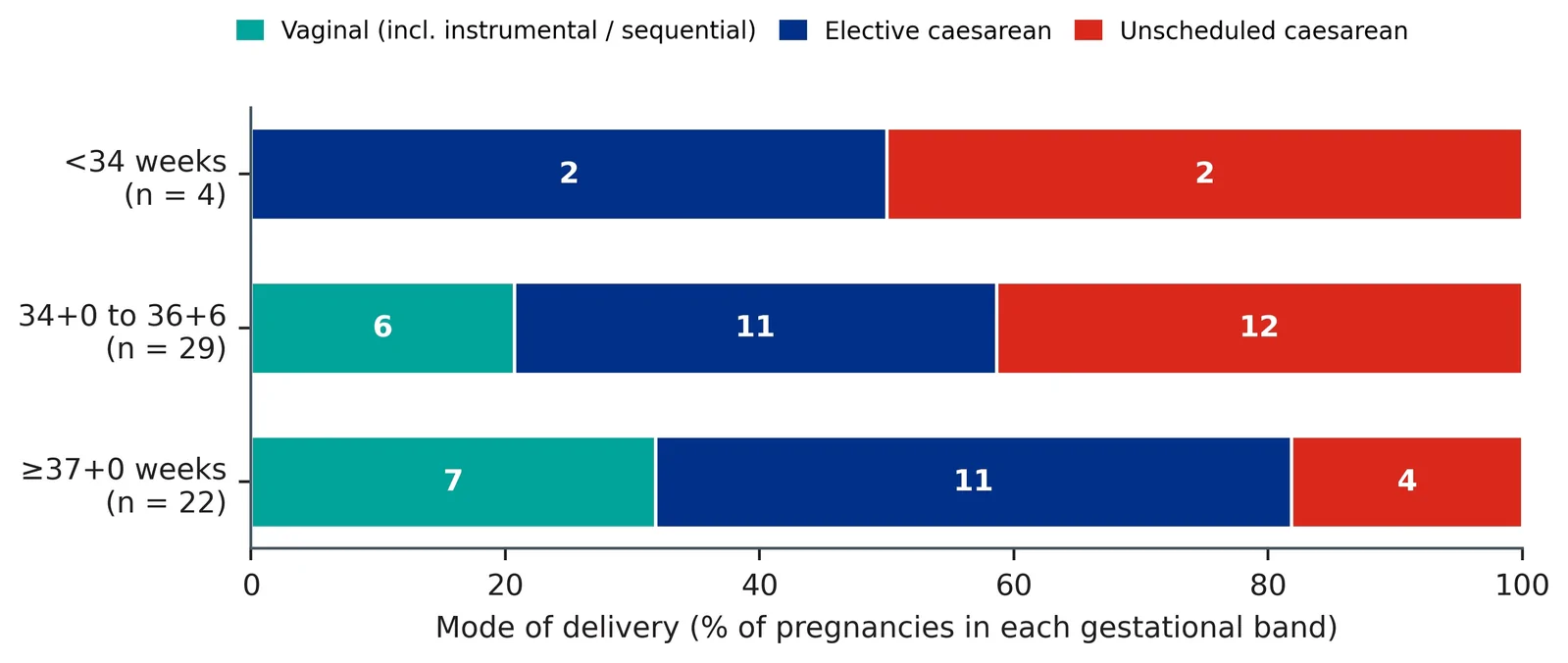
Mode of delivery by gestational age band (number of pregnancies)

Vaginal birth of the first twin was most common when both twins were cephalic (nine of 20, 45.0%) and rare when the first twin was non-cephalic (one of 17, 5.9%). All nine women (100%) with a previous cesarean had a repeat cesarean.

The first-listed indication for cesarean birth was recorded as maternal request in 10 of 42 cesareans (23.8%), malpresentation in six (14.3%), and previous cesarean in six (14.3%) (Table 4). The first-listed indication does not fully capture the clinical factors contributing to the decision: 16 of 17 women (94.1%) with a non-cephalic first twin and all nine (100%) with a previous cesarean had a cesarean, but only six of each were listed under those headings.

**Table 4:** Primary documented indication for cesarean birth of both twins (n = 42) The sequential delivery (cesarean for a retained second twin only) is excluded.

Indication	n (%)
Maternal request or choice	10 (23.8)
Malpresentation	6 (14.3)
Previous cesarean	6 (14.3)
Preterm labor or rupture of membranes	5 (11.9)
Fetal compromise (abnormal cardiotocography)	5 (11.9)
Growth restriction/placental dysfunction/abnormal Doppler	3 (7.1)
Hypertensive disorder/pre-eclampsia	3 (7.1)
Placental abruption (confirmed or suspected)	2 (4.8)
Failed induction of labor	1 (2.4)
Monochorionic twins alone	1 (2.4)

All 10 women whose cesarean was recorded as a maternal request had DCDA twins and planned cesareans at 36+0 to 37+5 weeks (median 37+1), and none had a previous cesarean. Seven of the ten (70.0%) had at least one non-cephalic twin (first twin non-cephalic in three; second twin only in four), one had gestational hypertension, and only two (20.0%) had both twins cephalic with no other recorded clinical factor. Other recorded factors were a fetal cardiac anomaly, gestational diabetes, polyhydramnios, assisted conception (one woman each), and maternal age ≥35 years (two women).

Non-cephalic presentation of the first twin was the main medical factor associated with cesarean birth. Cesarean occurred in 16 of 17 pregnancies (94.1%) with a non-cephalic first twin compared with 26 of 38 (68.4%) with a cephalic first twin (p = 0.045), and a non-cephalic second twin showed a similar pattern (24 of 27, 88.9%, vs 18 of 28, 64.3%; OR 4.33; 95% CI 0.93 to 28.01; p = 0.055) (Table 5). These associations largely reflect clinical practice, because a non-cephalic first twin or a previous cesarean usually leads to planned cesarean birth, and they should not be interpreted as independent risk factors.

**Table 5:** Factors associated with cesarean birth of both twins (n = 42) versus vaginal delivery of the first twin (n = 13) Fisher’s exact test. OR: conditional maximum likelihood estimate with exact 95% CI. Not estimable: OR is infinite because of a zero cell. Fisher's exact p-values and exact CIs are calculated differently and can disagree near p = 0.05; such results should be regarded as inconclusive. The identical rows for monochorionic twins and gestational diabetes reflect identical counts in different women (one woman had both) and were verified against the source data. OR: odds ratio, CI: confidence interval, BMI: body mass index, MCDA: monochorionic diamniotic

Factor	Cesarean n (%)	Vaginal n (%)	p	OR (95% CI)
Previous cesarean section	9 (21.4)	0 (0.0)	0.096	Not estimable (0.65-∞)
Maternal age ≥35 years	11 (26.2)	1 (7.7)	0.255	4.17 (0.50-198.31)
First twin non-cephalic	16 (38.1)	1 (7.7)	0.045	7.18 (0.90-335.04)
Second twin non-cephalic	24 (57.1)	3 (23.1)	0.055	4.33 (0.93-28.01)
Preterm delivery (<37 weeks)	27 (64.3)	6 (46.2)	0.334	2.07 (0.49-9.02)
Growth restriction/placental dysfunction/abnormal Doppler	9 (21.4)	3 (23.1)	1.000	0.91 (0.18-6.23)
MCDA twins	8 (19.0)	1 (7.7)	0.669	2.78 (0.31-135.57)
Gestational diabetes	8 (19.0)	1 (7.7)	0.669	2.78 (0.31-135.57)
Nulliparity	19 (45.2)	4 (30.8)	0.522	1.84 (0.43-9.50)
Assisted conception	8 (19.0)	2 (15.4)	1.000	1.29 (0.21-14.24)
Hypertensive disorder	7 (16.7)	2 (15.4)	1.000	1.10 (0.17-12.35)
Rupture of membranes before 37 weeks	4 (9.5)	2 (15.4)	0.619	0.59 (0.07-7.28)
Booking BMI ≥30 kg/m² (n = 37 with BMI)	12 (44.4)	2 (20.0)	0.260	3.11 (0.49-35.43)

Factors associated with preterm birth

All 12 pregnancies (100%) with growth restriction or abnormal Doppler findings were delivered before the recommended window, compared with 19 of 43 pregnancies (44.2%) without these findings (p < 0.001). Similarly, all six pregnancies (100%) with rupture of membranes before 37 weeks were delivered before the recommended window, compared with 25 of 49 pregnancies (51.0%) without this complication (p = 0.030). When chorionicity-specific windows were applied, MCDA pregnancies were not significantly more likely than DCDA pregnancies to deliver early (seven of nine, 77.8%, vs 24 of 46, 52.2%; p = 0.271), nor were pregnancies after assisted conception (eight of 10, 80.0%, vs 23 of 45, 51.1%; p = 0.159). The CIs were very wide (Table 6). These associations are partly expected by design, because growth restriction and prelabor rupture of membranes are themselves indications for earlier birth. Among the seven pregnancies with explicitly documented fetal or selective growth restriction, all were delivered before the recommended window.

**Table 6:** Factors associated with delivery before the chorionicity-specific recommended window (before 37+0 weeks for DCDA and before 36+0 weeks for MCDA twins; 31 of 55 women) Fisher p-values and exact CIs can disagree near p = 0.05; such results should be regarded as inconclusive. The identical rows for previous cesarean and gestational diabetes reflect identical counts in different women (two women had both) and were verified against the source data. OR: odds ratio, CI: confidence interval, DCDA: dichorionic diamniotic, MCDA: monochorionic diamniotic

Factor	Exposed (n)	Before window if exposed, n (%)	Before window if unexposed, n (%)	p	OR (95% CI)
Growth restriction/placental dysfunction/abnormal Doppler	12	12 (100.0)	19 (44.2)	<0.001	Not estimable (2.95-∞)
MCDA twins	9	7 (77.8)	24 (52.2)	0.271	3.15 (0.52-34.21)
Assisted conception	10	8 (80.0)	23 (51.1)	0.159	3.74 (0.65-40.00)
Rupture of membranes before 37 weeks	6	6 (100.0)	25 (51.0)	0.030	Not estimable (0.99-∞)
Hypertensive disorder	9	5 (55.6)	26 (56.5)	1.000	0.96 (0.18-5.51)
Maternal age ≥35 years	12	8 (66.7)	23 (53.5)	0.519	1.72 (0.39-9.03)
Nulliparity	23	15 (65.2)	16 (50.0)	0.286	1.85 (0.55-6.60)
Previous cesarean section	9	6 (66.7)	25 (54.3)	0.716	1.66 (0.31-11.54)
Gestational diabetes	9	6 (66.7)	25 (54.3)	0.716	1.66 (0.31-11.54)
Cervical cerclage and/or progesterone	8	5 (62.5)	26 (55.3)	1.000	1.34 (0.23-9.63)
Structural anomaly or variant	3	2 (66.7)	29 (55.8)	1.000	1.57 (0.08-97.59)
Polyhydramnios	3	1 (33.3)	30 (57.7)	0.575	0.37 (0.01-7.60)

Maternal outcomes

Median EBL was 700 mL (IQR 374 to 1100). Using the study definition, postpartum hemorrhage occurred in 19 women (34.5%; 95% CI 23.4-47.7): 15 of 42 (35.7%) after cesarean and four of 13 (30.8%) after vaginal delivery. Postpartum hemorrhage was recorded as a diagnosis in the clinical notes for 14 women (25.5%), all of whom met the study definition; the remaining five met the blood-loss threshold without a recorded diagnosis. Overall, 16 women (29.1%) lost ≥1000 mL and eight (14.5%; 95% CI 7.6-26.2) lost ≥1500 mL. Three women (5.5%) received a blood transfusion, three (5.5%) required ITU or HDU care, four (7.3%) were readmitted, and no woman died (Table 7). Blood loss was higher after cesarean than after vaginal delivery (median 836 mL versus 350 mL; p = 0.002), and composite maternal morbidity affected 17 of 42 women (40.5%) after cesarean compared with one of 13 (7.7%) after vaginal delivery (p = 0.041). Composite morbidity largely reflected blood loss: 16 of the 18 women met the EBL ≥1000 mL criterion, and two qualified through transfusion or ITU/HDU care alone. When blood loss was excluded, transfusion or ITU/HDU care occurred in six women (10.9%; 95% CI 5.1-21.8): five of 42 (11.9%) after cesarean and one of 13 (7.7%) after vaginal birth (p=1.000). It occurred after nine of 24 elective (37.5%) and eight of 18 unscheduled (44.4%) cesareans. The comparison with vaginal birth is likely to be confounded by indication, because women who gave birth vaginally were a selected, lower-risk group, and blood loss may be estimated differently after cesarean and vaginal birth.

**Table 7:** Maternal outcomes (N = 55) * Vaginal delivery of the first twin, including one forceps-assisted and one sequential delivery. Composite: EBL ≥1000 mL, transfusion, or ITU/HDU admission. Dashes: not tabulated by mode. ITU: intensive treatment unit, HDU: high-dependency unit, IQR: interquartile range, EBL: estimated blood loss

Outcome	All (N = 55)	Cesarean (n = 42)	Vaginal* (n = 13)
EBL, mL, median (IQR)	700 (374-1100)	836 (497-1268)	350 (157-662)
Postpartum hemorrhage, study definition†, n (%)	19 (34.5)	15 (35.7)	4 (30.8)
Postpartum hemorrhage recorded as a diagnosis in the notes, n (%)	14 (25.5)	13 (31.0)	1 (7.7)
EBL ≥500 mL, n (%)	35 (63.6)	31 (73.8)	4 (30.8)
EBL ≥1000 mL, n (%)	16 (29.1)	15 (35.7)	1 (7.7)
EBL ≥1500 mL, n (%)	8 (14.5)	8 (19.0)	0 (0.0)
Blood transfusion, n (%)	3 (5.5)	2 (4.8)	1 (7.7)
ITU or HDU admission, n (%)	3 (5.5)	3 (7.1)	0 (0.0)
Composite maternal morbidity, n (%)	18 (32.7)	17 (40.5)	1 (7.7)
Transfusion or ITU/HDU admission (composite without blood loss), n (%)	6 (10.9)	5 (11.9)	1 (7.7)
Length of stay, days, median (IQR)	4 (3-6)	4 (3-6)	4 (3-7)
Readmission, n (%)	4 (7.3)	-	-
Maternal death, n (%)	0 (0.0)	0 (0.0)	0 (0.0)

Neonatal outcomes

Among 107 liveborn infants with data, mean birthweight was 2417 ± 457 g, and 57 infants (53.3%) weighed <2500 g. Among the 53 pairs with two liveborn infants, the mean birthweight was 2438 ± 456 g for the first twin and 2394 ± 464 g for the second (mean difference 44 g; 95% CI −54 to 142 g; paired t-test t = 0.90, df = 52, p = 0.373). The median birthweight discordance was 6.4%; nine of 53 pairs (17.0%; 95% CI 9.2-29.2) had ≥20% discordance, and seven (13.2%) had ≥25% discordance (Figure 4).

**Figure 4:**
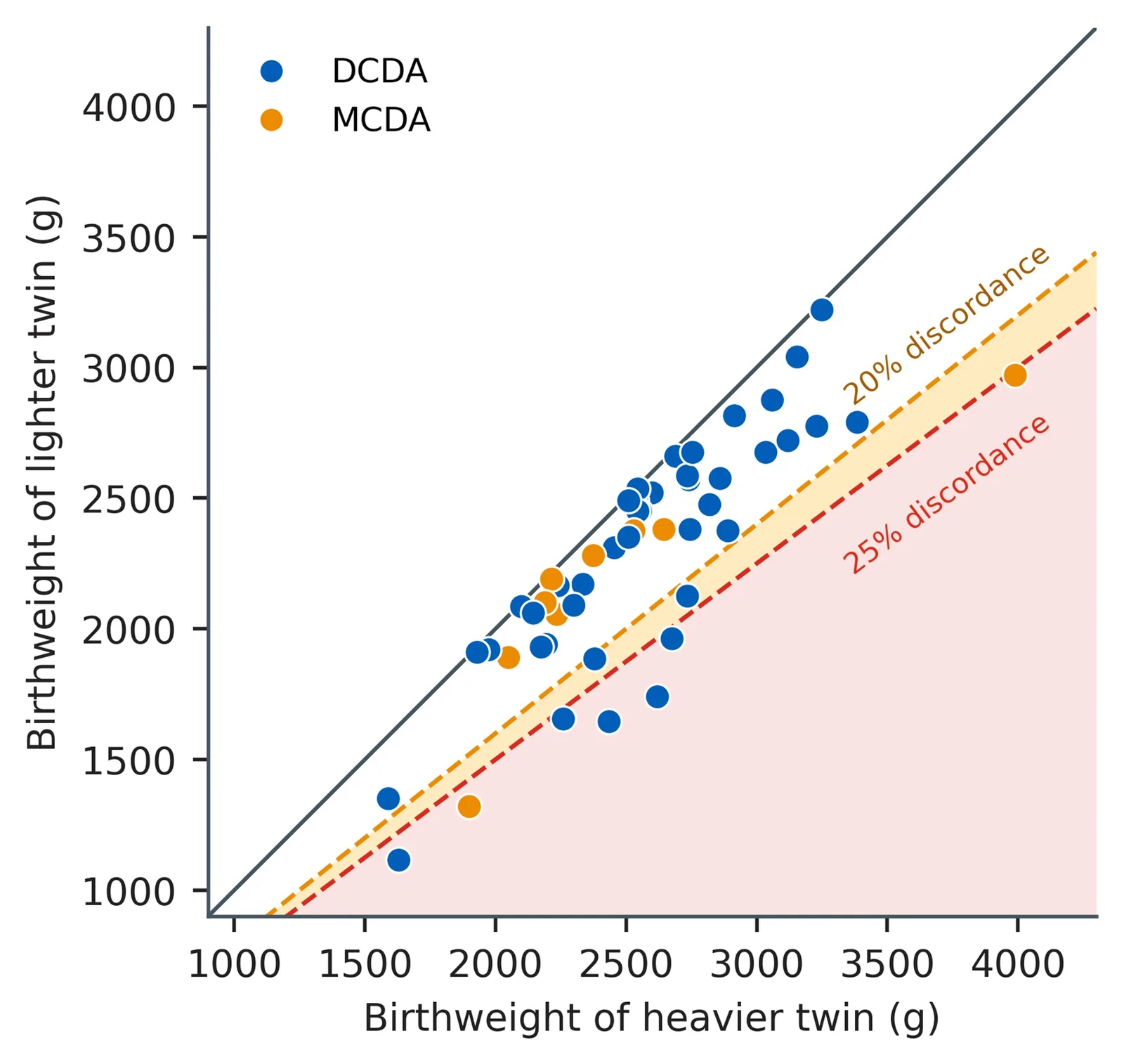
Birthweight of the lighter versus heavier twin (53 pairs with two liveborn infants). The solid line shows equal birthweights; dashed lines mark 20% and 25% discordance DCDA: dichorionic diamniotic, MCDA: monochorionic diamniotic

Growth restriction was defined from antenatal documentation rather than birthweight (see Definitions). Seven of the nine discordant pairs (77.8%) had an antenatally documented growth concern: six were recorded in the antenatal record as fetal or selective growth restriction or abnormal umbilical artery Doppler, and one as small for gestational age at delivery. Antenatal ultrasound discordance in estimated fetal weight, recorded at the last scan for 51 of 53 pairs (96.2%), correlated moderately with birthweight discordance (Spearman ρ = 0.48; p < 0.001) but was ≥20% in only four of the nine discordant pairs (44.4%). The two measures may differ because growth restriction can be diagnosed based on a low estimated fetal weight or abnormal Doppler findings, even when the estimated weight difference between twins is below 20%, and because the interval between the last scan and birth was not recorded. Taken together, eight of the nine discordant pairs (88.9%) had either a documented growth concern or scan discordance ≥20%.

Umbilical cord pH (sample type not recorded) was <7.10 in four of 85 infants (4.7%), and the five-minute Apgar score was <7 in two of 107 (1.9%) (Table 8).

**Table 8:** Neonatal outcomes Percentages use the denominator shown for each section or row. Whether cord samples were arterial or venous, and the level of care and indication for neonatal unit admission, were not recorded. Fetal death is classified as a fetal loss before 24 weeks, not a stillbirth (see text). Complications were briefly recorded in the final dataset and are likely to be underascertained, so their percentages are minimum estimates. SD: standard deviation

Outcome	n	%
Per liveborn infant (n = 107 with data)		
Birthweight, g, mean ± SD	2417 ± 457	
Birthweight <2500 g	57	53.3
Birthweight <1500 g	3	2.8
Birthweight <1000 g	0	0.0
Cord pH, mean ± SD (n = 85; sample type not recorded)	7.275 ± 0.085	
Cord pH <7.10 (n = 85)	4	4.7
Apgar <7 at 5 min	2	1.9
Apgar <7 at 1 min	13	12.1
Per twin pair (n = 53 pairs)		
Birthweight, first twin, g, mean ± SD	2438 ± 456	
Birthweight, second twin, g, mean ± SD	2394 ± 464	
Mean difference, g (95% CI); paired t-test	44 (−54 to 142); p = 0.373	
Birthweight discordance ≥20%	9	17.0
Birthweight discordance ≥25%	7	13.2
Birthweight discordance ≥30%	4	7.5
Antenatal scan discordance ≥20% (n = 51 pairs)	5	9.8
Per pregnancy: at least one twin (n = 54)		
Neonatal unit admission (any level of care)	20	37.0
Respiratory distress syndrome	3	5.6
Suspected or confirmed sepsis	4	7.4
Neonatal jaundice	2	3.7
Neonatal anemia	1	1.9
Birth injury (Erb’s palsy)	1	1.9
Mortality		
Fetal death confirmed at 23+3 weeks, delivered at 37+5 weeks (n = 110 fetuses)	1	0.9
Neonatal death (n = 109 live births)	0	0.0

At least one twin was admitted to the neonatal unit, at any level of care, in 20 of 54 pregnancies (37.0%; 95% CI 25.4-50.4), including all four pregnancies (100%) delivered before 34 weeks. Admission was less frequent at later gestations, occurring in 11 of 29 pregnancies (37.9%) delivered at 34+0 to 36+6 and in five of 21 (23.8%) delivered at 37 weeks or later (Table 8). The level of care and the indication for admission were not recorded. One fetus (0.9%) died in utero at 23+3 weeks; there were no neonatal deaths among 109 live births (0.0%). Table 9 shows these results alongside UK national audit and Norwegian registry data.

**Table 9:** Selected outcomes in the present study alongside UK and other high-income data The NMPA preterm figure refers to all multiple births, of which 98.6% were twins; other NMPA figures refer to twins. Comparator values are shown as published. No formal statistical comparison was made, and a comparator value lying within our 95% CI should not be interpreted as evidence that outcomes are equivalent. Outcomes with very few events (sequential births, low Apgar score, mortality) are not shown because comparison would be uninterpretable. Definitions vary across sources. -: not reported in the cited source, NMPA: National Maternity and Perinatal Audit, CI: confidence interval, UK: United Kingdom

Measure	Present study, n (%; 95% CI)	NMPA, Great Britain, 2023 (8301 women) [1]	Norwegian registry, 1999-2014 (16174 twin pregnancies) [3]
Preterm birth <37 weeks	33 (60.0; 46.8-71.9)	64% of all multiple births	-
Cesarean birth of both twins	42 (76.4; 63.7-85.6)	79.4% (42.9% planned, 36.5% unplanned)	-
Vaginal birth of both twins (unassisted or instrumental)	12 (21.8; 12.9-34.4)	17.9% (13.3% unassisted, 4.55% instrumental)	-
Blood loss ≥1500 mL	8 (14.5; 7.6-26.2)	11.6%	-
Pre-eclampsia	5 (9.1; 3.9-19.6)	-	11.8%

## Discussion

In this nine-month, two-site series of 55 twin deliveries, preterm birth, cesarean birth, postpartum hemorrhage, and neonatal unit admission were common, and short-term survival was good. Point estimates for preterm and cesarean birth were close to UK national audit figures [1], but with 55 women the 95% CIs were wide, so this study cannot show whether local outcomes are similar to or different from national data (Table 9).

Timing of birth

Preterm birth affected 33 of 55 pregnancies (60.0%; 95% CI 46.8-71.9), compared with about 64% nationally [1]. Because NICE recommends planned birth from 36+0 weeks for uncomplicated MCDA twins and from 37+0 weeks for uncomplicated DCDA twins [6], delivery before 37 weeks is expected by design in MCDA pregnancies, and the conventional preterm threshold is not a meaningful outcome in this group. Assessed against chorionicity-specific windows, seven of nine MCDA pregnancies (77.8%) delivered before 36+0 weeks, and only two delivered within the 36+0 to 36+6 window. Six of the seven early MCDA deliveries had a recorded maternal or fetal indication for early birth; the seventh, a planned cesarean at 35+1 weeks for a breech first twin, had no recorded reason for delivery before 36+0 weeks. Early delivery in MCDA pregnancies therefore warrants further local review. Among uncomplicated pregnancies, 13 of 19 (68.4%) delivered within the recommended windows.

Mode of birth

The cesarean rate was 76.4% (42 of 55; 95% CI 63.7-85.6), and the national figure of 79.4% [1] falls within this interval. The planned mode of birth was not recorded, so the success of planned vaginal birth could not be assessed against Twin Birth Study benchmarks [7]. The first-listed indication was recorded as a maternal request for 10 cesareans, but seven of these ten women had a non-cephalic twin, one had gestational hypertension, and only two had no other recorded clinical factor. Non-cephalic presentation of the second twin is a recognized reason for counseling about cesarean birth, so these cesareans should not be interpreted as non-medical requests. Because only the first-listed indication was available, and planned mode and counseling were not recorded, our data cannot assess the appropriateness of cesarean decisions; recording the planned mode of birth and all contributing reasons would allow this in future audits.

Maternal morbidity

Postpartum hemorrhage occurred in 19 women (34.5%) by the study definition, but only 14 had a recorded diagnosis, which suggests under-recording in the clinical notes. Blood loss of ≥1500 mL occurred in eight women (14.5%; 95% CI 7.6-26.2); the national figure of 11.6% [1] lies within this interval, but with eight events, no formal comparison is possible. Blood loss and composite morbidity were higher after cesarean (17 of 42, 40.5%, vs one of 13, 7.7%), although estimates were imprecise and EBL measurement varies by delivery mode. Without a singleton comparator, this study cannot quantify the excess risk associated with twin pregnancy; RCOG guidance already identifies multiple pregnancy as a risk factor for postpartum hemorrhage [13].

Neonatal outcomes and growth surveillance

The absence of neonatal deaths among 109 live births does not indicate better-than-expected neonatal outcomes; with this sample, the upper 95% confidence limit for neonatal mortality was 3.4%. At least one twin was admitted to the neonatal unit in 20 of 54 pregnancies (37.0%). Because the level of care and the indication for admission were not recorded, this figure combines short admissions for minor problems with intensive care, and it should not be interpreted as a measure of serious neonatal morbidity. Birthweight discordance of ≥20% affected nine of 53 pairs (17.0%) and is clinically important because it is associated with perinatal risk [15,16]. Eight of these nine pairs had either an antenatally documented growth concern or scan discordance ≥20%, but scan discordance alone identified only four. Recording the date of the last scan would allow future audits to account for the scan-to-birth interval.

Data quality

Chorionicity was ascertained for all 55 women (100%), but through manual review of the clinical record rather than structured coding, so this does not show that the electronic record captures chorionicity better than national datasets, in which it is coded for fewer than 7% of multiple births [1]. Booking BMI was missing for 18 women (32.7%), and planned mode of birth, cesarean indications beyond the first listed, cord sample type, neonatal level of care, and admission indication were not recorded. Adding these fields to the electronic record would make future audits more reliable.

Strengths and limitations

This study included all twin deliveries identified during the first nine months of the electronic record, with transparent coding rules and internal checks, and chorionicity was ascertained for every pregnancy. The study is limited by its small sample size and single-trust, retrospective cross-sectional design. There was no denominator of twin pregnancies at risk, no singleton comparator, and no fixed follow-up after the birth admission. Women transferred antenatally to give birth elsewhere, including complex monochorionic pregnancies referred for tertiary fetal medicine care, were not captured, which may bias the series towards lower risk. Estimates are imprecise, rare outcomes cannot be assessed, and the many unadjusted comparisons increase the risk of chance findings, so statistically significant associations should be regarded as hypothesis-generating only.

Several associations reflect clinical practice rather than independent risk factors: growth restriction and prelabor rupture of membranes indicate earlier birth, and a non-cephalic first twin or a previous cesarean usually leads to cesarean birth. The comparison of maternal morbidity between cesarean and vaginal birth is confounded by indication; elective and unscheduled cesareans were pooled, and the composite outcome largely restates blood loss, which may be estimated differently by mode of birth. Outcome misclassification is possible because a single abstractor coded free-text entries, growth restriction was a broad composite assigned retrospectively, only the first-listed cesarean indication was available, and neonatal complications are likely underascertained. Preterm births were classified as spontaneous or provider-initiated from the documented indication only, because onset of labor was not a structured field. Cord sample type, neonatal level of care and admission indication, planned mode of birth, the method of blood-loss measurement, the gestational dating method, total births, and site identifiers were not recorded. The literature comparison was narrative and indicative only, and the findings may not be generalizable beyond this service. Future work should use a larger, multi-year, multi-site dataset with structured fields to support adjusted analyses.

## Conclusions

Preterm birth, cesarean birth, postpartum hemorrhage, and neonatal unit admission were common in this series of twin deliveries, and short-term survival was good. With 55 women, CIs were wide, and the study cannot show whether outcomes differ from national data. Early delivery of MCDA twins relative to the NICE window and the reasons recorded for cesarean birth warrant local review. Structured documentation of the planned mode of birth and the reasons for the decision, cesarean urgency, neonatal level of care, and admission indication would support a more reliable audit. The associations reported are exploratory and do not imply causation.

## Appendices

**Table 10:** Appendix 1: data abstraction pro forma (33 fields) IVF: in vitro fertilization, DCDA: dichorionic diamniotic, MCDA: monochorionic diamniotic, MCMA: monochorionic monoamniotic, HDU: high-dependency unit, ITU: intensive treatment unit, BMI: body mass index

No.	Field (as recorded)
1	Hospital number (used only to locate the record; not analyzed)
2	Maternal age (years)
3	Parity
4	Previous cesarean section (yes/no)
5	Booking BMI
6	Ethnic group
7	Mode of conception (natural/IVF/other)
8	Gestation at booking (weeks)
9	Smoking (yes/no)
10	Chorionicity (DCDA/MCDA/MCMA)
11	Maternal medical and obstetric complications (free text)
12	Twin-related complications (free text)
13	Cervical cerclage/progesterone/none
14	Ultrasound growth discordance at last scan (%)
15	Gestation at delivery (weeks+days)
16	Mode of delivery, including cesarean category
17	Indication for delivery (free text)
18-19	Presentation of first and second twin
20	Estimated blood loss (mL)
21	Delivery complications (free text, including hemorrhage, transfusion, HDU/ITU)
22	Length of admission (days)
23	Readmission (yes/no)
24-25	Birthweight of first and second twin (g)
26	Birthweight discordance (%)
27-28	Apgar scores of first and second twin
29-30	Umbilical cord pH of first and second twin
31-32	Sex of first and second twin
33	Neonatal complications (free text, including neonatal unit admission)
